# Attitudes about Cigarette Smoking, Perceived Consequences of Smoking, and Seeking Assistance with Cessation among Black and White Cigarette Smokers: A Qualitative Study

**DOI:** 10.1155/2023/9298027

**Published:** 2023-05-20

**Authors:** Alina Shevorykin, Lesia M. Ruglass, Roberta Freitas-Lemos, Alexandria G. Bauer, Shannyl Baez, Christine E. Sheffer

**Affiliations:** ^1^Roswell Park Comprehensive Cancer Center, USA; ^2^The City College of New York, City University of New York, USA; ^3^Center for Alcohol and Substance Use Studies, Rutgers University, USA; ^4^Fralin Biomedical Research Institute at Virginia Tech Carilion, USA

## Abstract

**Introduction:**

Research has identified significant racial differences in cigarette smoking behavior, associated disease risk, likelihood of cessation, and mortality from smoking-related diseases. The current study assessed, via qualitative narrative analysis, racial differences in participants' motivations for smoking, perceived consequences of smoking, and how participants deal with cravings/withdrawal, as well as thoughts and feelings about quitting, seeking assistance with quitting, and the importance of social support in quitting.

**Methods:**

Semistructured interviews were conducted with 11 Black and 11 White cigarette smokers. Data were analyzed using the Thematic Networks Analysis process, which entailed coding the data and constructing thematic networks by identifying basic and organizing themes.

**Results:**

While there were no descriptive racial differences identified in participants' motivation for smoking or perceived consequences of smoking, differences existed between Blacks and Whites in terms of approaches in dealing with smoking cravings and withdrawal, perceived self-efficacy in controlling cravings, preferred methods of learning about and receiving smoking cessation assistance, and overall preference for receiving cessation-related support.

**Conclusions:**

Further investigation is needed into racial differences in methods to deal with cigarette cravings and withdrawal, preferences for receiving cessation information, and social support for cessation. This research will further develop our understanding of and ability to address factors underlying racial disparities in smoking behavior and cessation, as well as inform the development of future smoking cessation interventions.

## 1. Introduction

Cigarette smoking is the leading cause of disease, disability, and death in the United States, accounting for over 480,000, or one in every five, deaths each year [1]. Despite significant reductions in smoking prevalence in the past 50 years, in 2019, an estimated 49.1 million adults in the US reported using at least one tobacco product [2]. Cigarette smoking significantly contributes to racial disparities in tobacco-related mortality and disease, including significantly greater rates of cardiovascular disease and cancer among Blacks [3] compared to Whites. Although Black individuals initiate cigarette smoking at a later age [4], smoke fewer cigarettes per day [4], and make more quit attempts than White individuals [4–6], Black individuals are significantly less likely to achieve and maintain long-term abstinence from smoking [5].

Numerous intersecting individual, community, and environmental factors are associated with racial disparities in smoking cessation [7], including differences in smoking behaviors [8]; attentional bias to smoking cues [9]; use of menthol tobacco products [6]; enforcement of tobacco control policies, such as smoke-free air laws, youth access laws, and tobacco price [10, 11]; exposure to tobacco advertising [12]; and socioeconomic disparities [13, 14]. Differences in support from medical providers are also evident, with Whites being more than twice as likely to be advised by their doctor to quit smoking than Blacks [6]. In addition, there are significant racial differences in expectations about withdrawal, challenges with quitting, the effectiveness of smoking cessation medications, and the efficacy of quit attempts [15]. Black men also tend to report lower knowledge levels of smoking cessation treatment options and lower self-efficacy for quitting smoking [15].

Public health strategies such as advertising and increased price of cigarettes have increased willingness and intentions to quit among Black smokers, but this has not been translated into improvements in cessation [16, 17]. These issues are compounded by the fact that Black individuals are more likely to be excluded from large smoking cessation trials [18] and existing evidence-based treatments for tobacco dependence are less effective for Black than White individuals [19], particularly for long-term and more highly dependent individuals who smoke [20]. While the development of tailored interventions for Black individuals who smoke are on the rise and remarkable gains relative to White individuals for up to 3 months postquit have been observed, Black individuals show precipitous long-term relapse rates and ultimately no increased benefit from standard approaches [14]. At present, there is no clear understanding of why this might be the case.

While quantitative analyses have explored racial differences in the mechanisms underlying tobacco-related disparities, such as abstinence-related expectancies around withdrawal effects, self-efficacy, and motivation to quit [15], as well as consequences of smoking [21], perceptions of and strategies for managing cravings and withdrawal [22], and thoughts or feelings about quitting and seeking assistance [23, 24], a qualitative investigation of these factors has not yet contributed to the evidence. While quantitative research is critical to establishing and testing relationships and hypotheses, qualitative research is essential to clarifying observed phenomena in natural, rather than experimental settings, as well as to establishing theories and hypotheses and describing processes such as decision-making and attitudes [25]. This study is aimed at providing an initial understanding of the attitudes associated with smoking and smoking cessation and helping identify potential areas for further investigation regarding racial differences. Understanding smoking-related attitudes and possible differences between Black and White individuals who smoke is a vital first step to evaluating the extent of these differences and how they might impact treatment and eventually translate to new therapeutic approaches [26]. Narrative analysis is one of the most widely used forms of qualitative analysis, and it has been used successfully to identify factors affecting individuals' success in smoking cessation, including the importance of perceived self-efficacy, expectations of treatment effectiveness, and ambivalence about change [26, 27]. To our knowledge, this is the first qualitative investigation of consequences of smoking, perceptions of and strategies for managing cravings and withdrawal, and thoughts or feelings about quitting and seeking assistance.

## 2. Methods

### 2.1. Participants

Participants were non-Hispanic adults aged 18 years or older who were identified as Black or African American and White, smoked cigarettes daily (verified by an exhaled breath carbon monoxide level of 5 ppm or above), reported no current or past psychiatric disorders, passed a 10-panel urine toxicology test for drugs of abuse, and drank no more than 7 alcoholic drinks per week for females and no more than 14 drinks per week for males. Participants were recruited by online advertisements and flyers posted in the Harlem neighborhood, near the City College of New York campus. This study was approved by the Institutional Review Board of the City University of New York. All participants provided written consent. Participants were compensated $35 for their time and effort and received a roundtrip MetroCard for transportation.

### 2.2. Materials and Procedure

Participants who were identified as non-Hispanic Black were recruited first. Non-Hispanic White participants were then recruited to match Black participants on gender, age, years of education, and number of cigarettes smoked per day. Demographic information was collected, including partnered status, annual household income per year, age of initiation of cigarette smoking, years of smoking, motivation to quit smoking, and number of quit attempts in the past year. Nicotine dependence level was assessed using the Fagerstrom Test for Nicotine Dependence [28].

The development of the structured interview used to assess participants' attitudes about smoking and smoking cessation was guided by the published literature on racial differences in smoking behaviors, access to treatment for smoking cessation, and environmental factors associated with smoking cessation [3, 17, 26, 29–33]. The structured interview was piloted with six preliminary participants (3 Black and 3 White). Participant feedback from the pilot interviews was used to revise interview questions for clarity, change the order of some questions, and add follow-up questions.

The final structured interview included 5 topics comprised of 17 questions organized as follows: *consequences of smoking*: “What are the reasons you smoke?”, “What are the effects of smoking for you?”, “What are the benefits of smoking for you?”, and “What are the risks of smoking for you?”; *craving and withdrawal*: “What thoughts and/or feelings do you associate with cigarette cravings?”, “What do you do when it's impossible to smoke?”, and “When you don't smoke for a while, how do you start thinking or feeling?”; *smoking cessation*: “When you think about quitting, how do you feel?” and “What challenges do you associate with quitting smoking?”; *help with smoking cessation*: “How easy is it for you to find assistance with quitting smoking?”, “What type of assistance do you think would be effective?”, “If you were looking for more information on how to quit smoking, what would be the best way to get you that information?”, “What is preventing you from seeking assistance in quitting?”, and “There are a lot of different ways we can provide information on smoking cessation, for example, written materials, group discussion, and talking with your doctor. What do you think would suit you best?”; and *social support*: “What kind of support would you need from your social environment to help you quit smoking?”, “What kind of help would you need from people in your life in order to quit?”, and “How do relationships help or hurt your quit attempts?”.

Interviews lasted between 60 and 90 minutes and were conducted by masters-level graduate students who successfully completed an in-depth two-day training on qualitative interviewing. Questions were open-ended and intended to prompt in-depth responses. Interviewers were allowed to probe participants to ensure the responses were as complete as possible. All interviews were transcribed. Transcriptions were reviewed for accuracy.

### 2.3. Data Analysis

The data was analyzed using the Thematic Networks Analysis (TNA) process, which entails coding the data and constructing thematic networks by identifying basic and organizing themes [34]. Basic themes are used to group responses by similar viewpoints. Organizing themes are used to group basic themes into organizing concepts across multiple viewpoints.

Consistent with recommendations, interview transcripts were independently analyzed and coded by two researchers trained in the TNA process. First, the researchers independently highlighted all content in the transcripts that comprised responses to specific questions. Researchers' highlights were compared to ensure the researchers consistently identified content that qualified as responses to specific questions. Then, for each highlighted response, researchers independently identified and documented keywords. The keywords were then organized by basic themes. No differences were found between the researchers in the identification of keywords and basic themes. The researchers then collaborated to identify organizing themes.

## 3. Results

### 3.1. Demographics

Participants (*n* = 22) included 11 individuals who were identified as Black and 11 who were identified as White, all non-Hispanic, 90% of whom were men. The average age was 48 years (SD = 8.97). They were a couple of notable racial difference descriptively: White participants (*M*_age_ = 15, SD = 2.32) start to smoke cigarettes earlier than Black participants (*M*_age_ = 19.36, SD = 4.08); more of Black participants (27.3%) were in long-term relationship than White participants (18.2%); and White participants were split in between being motivated to quit smoking (45%) and not motivated or not at all motivated (45%), where majority of Black participants (45%) were motivated to quit smoking and not motivated or not at all motivated (36%). In terms of means and standard deviations, both groups were similar in age, education, cigarettes per day, years of smoking, nicotine dependence level, and number of quit attempts in the past year (see Table 1).

### 3.2. Results

The TNA is summarized in Table 2 and discussed in detail by topic below, organized by question set:

#### 3.2.1. Consequences of Smoking

Nine basic themes emerged from the responses to the questions about the consequences of smoking and included the following: (1) coping (e.g., “takes the edge off,” “takes away stress and tension, anxiety,” “I find it relaxing,” and “calms my nerves”), (2) enjoyment (e.g., “I like to do it” and “I like the way they make me feel when I smoke”), (3) dependence or habit (e.g., “used to it,” “been smoking for 20 years,” and “it takes the craving out of me”), (4) social pressure/connectedness (e.g., “everybody was doing it and I thought it was cool,” “peer pressure and wanting to fit in with the older kids,” and “wanted to be part of the crowd, I guess”), (5) taste (e.g., “like the way it tastes” and “taste, I actually like it, the brand I smoke”), (6) relaxation (e.g., “it just makes me relax,” “its soothing at times,” and “It calms me down”), (7) financial burden (e.g., “it's a burden on finances” and “being broke”), (8) smell and appearance (e.g., “it smells…it sorta ruins my appetite,” “the smell is disheartening,” “people don't like the smell,” “yellow teeth,” “shortness of breath,” and “bad smell”), and (9) health concerns (e.g., “you pollute your body,” “affects your lungs, short of breath,” “it deteriorates my lungs,” “heart disease,” “emphysema,” “COPD,” “lung cancer,” and “throat cancer”). One organizing theme was identified for this topic: *the consequences of smoking cigarettes include benefits and risks*.

#### 3.2.2. Craving and Withdrawal

Six basic themes emerged from the responses to the questions about thoughts, feelings, and behaviors associated with craving and withdrawal, including the following: (1) negative feelings and discomfort (e.g., anxiety, stress, anger, irritation, aggravation, “I get crazy,” “mind is kind of racing,” “sweating,” and “lip biting”), (2) positive feelings (e.g., “opening up a new pack and that smell of the cigs before you light them up, I don't know I just like that” and “I'd like to take a puff, exhale it, feel the drag, take a nice drag”), (3) impulsive action (e.g., “I just do it, I don't think about it” and “impatience”), (4) use of cognitive distractions (e.g., “take my mind off it,” “accept it,” “mentally prepare myself,” “divert my thoughts,” “don't really think about it,” and “I put it out of my mind”), (5) use of behavioral distractions (e.g., “read, go on Facebook…,” “relax and say my prayers,” “find something else to do,” “I bite my lip a lot,” “sleep…count the minutes,” and “play games on my phone, whatever”), and (6) belief they can ignore/handle cravings without distraction (e.g., “nothing. just bear with it,” “I can control it,” and “It does not bother me like that these days”). Two organizing themes were identified for this topic: *craving and withdrawal generate approach and avoidance behaviors and the management of craving and withdrawal is a common experience.*

#### 3.2.3. Smoking Cessation

Six basic themes emerged from the responses to the questions about smoking cessation, including the following: (1) positive feelings (e.g., happy, hopeful, and confident), (2) negative feelings (e.g., anxiety, stress, weakness, and depression), (3) ambivalence (e.g., expressing positive and negative feelings concurrently), (4) physical dependence (e.g., “body is used to it” and “I give in to my cravings”), (5) lack of motivation (e.g., “I'm just not putting any effort into it”), (5) no substitutes (e.g., “what to put in its place afterwards” and “what is there to prevent me…nothing”), and (6) lack of interest (e.g., “never thought about quitting” and “nothing beats having that first cup of coffee and a cig”). One organizing theme was identified for this topic: *smoking cessation is characterized by significant cognitive dissonance.*

#### 3.2.4. Help with Smoking Cessation

Eight basic themes emerged from the responses to the questions about help with smoking cessation, including the following: (1) finding assistance is easy (e.g., “oh it's very easy…everybody… insurances want you to quit,” “if you want it, it's there,” and “I think it's fairly easy”), (2) finding assistance is difficult (e.g., “it's not easy because of the lifestyle I'm living right now” and “I don't have many friends that I could count on to go through the experience with me”), (3) awareness of various types of assistance (e.g., “patch,” “gum,” “counseling,” “support groups,” and “moral support from family and friends”), (4) no support needed (e.g., “[I] don't really want no assistance”), (5) ambivalence about seeking help (e.g., “my unwillingness to want to change right now to stop smoking”), (6) preference for written materials (e.g., “written instructions,” “written material if anything,” and “written materials...online”), (7) preference for speaking with a doctor (e.g., “talking with a doctor, cause it can prescribe stuff” and “probably talking to a doctor…I trust doctors”), and (8) preference for participating in group discussion (e.g., “maybe a group discussion” and “group...like to talk and hear other people's viewpoints”). Three organizing themes were identified for this topic: *knowledge about where to seek help for smoking cessation is common, individuals have strong preferences about what kind of help they prefer, and ambivalence about cessation prevents seeking help.*

#### 3.2.5. Social Support

Four basic themes emerged from the responses to the questions about social support related to smoking cessation, including the following: (1) need for a smoke-free environment (e.g., “I would need for them not to smoke in front of me,” “I would need to be around more positive people,” and “just to hang out with people who don't smoke”), (2) relationships hurt quit attempts (e.g., “they probably don't help” and “mostly hurt…it makes it harder”), (3) relationships help quit attempts (e.g., “by supporting me...be there for me” and “she helped...she wanted the best for me”), and (4) relationships neither hurt nor help quit attempts (e.g., “haven't hurt or helped” and “neither…it's all on me”). One organizing theme was identified for this topic: *social influences can be helpful or can be a barrier to smoking cessation.*

#### 3.2.6. Racial Differences in Basic Themes

Several basic themes appeared more frequently in the narratives of one racial group compared with the other. For *craving and withdrawal*, more White participants (*n* = 4) than Black participants (*n* = 1) reported that they can ignore or control cravings without using distractions. For *help with smoking cessation*, more Black participants (*n* = 6) than White participants (*n* = 1) preferred written materials, and more White participants (*n* = 5) than Black participants (*n* = 4) preferred speaking with a medical provider, while more White participants (*n* = 4) preferred participating in group discussion than Black (*n* = 2). For *social support*, more Black participants (*n* = 6) reported that relationships neither hurt nor helped their quit attempts than Whites (*n* = 3); and more Black participants (*n* = 4) said that relationships helped than White participants (*n* = 3). More Whites (*n* = 5) said that relationship hurts their quit attempts than Blacks (*n* = 1). There were no differences between groups among the basic themes for consequences of smoking or smoking cessation (see Table 3 for a summary of group differences).

## 4. Discussion

This study is aimed at providing an initial understanding of racial differences in the attitudes associated with smoking and cessation, as well as identifying potential areas for further investigation about racial differences in these attitudes. While quantitative analyses have previously explored racial differences in predefined quantitative factors [8], as well as the consequences of smoking [21], perceptions of and strategies for managing cravings and withdrawal [22], and thoughts or feelings about quitting and seeking assistance [23, 35], a qualitative investigation of these factors has not been previously undertaken.

The results showed that White participants reported being able to ignore or control cravings without the need for distraction more often than Black participants. This finding is consistent with the previous research indicating higher overall rates of craving reported by Blacks compared to Whites, suggesting that the former may perceive craving as a more pervasive problem [36]. There are multiple possible explanations for these racial differences in craving. From a biological perspective, differences in genetic predisposition to persistent smoking due to specific dopamine-related genes associated with addiction and craving have been found. Indeed, one study [37], in a sample of 88 Black individuals who smoke, found greater cue-induced craving among carriers of the D2 dopamine receptor gene, while another study highlighted the existence of large interpopulation differences in the frequency of the Taq1 alleles [38]. From an environmental perspective, greater exposure to cigarette cues, due to the increased tobacco advertising in minority neighborhoods [39, 40] could be associated with increased level of craving in Black participants [41]. From a social perspective, continuing experiences with race-based discrimination may affect brain functioning and psychophysiological responses in a way that results in negative health outcomes [42]. Racial differences in craving could be part of a self-regulation strategy to cope with stress [43]. Our findings suggest the need for further investigation of factors contributing to the ability to control cravings among smokers, and potential racial differences in these factors, given the importance of craving control to successful smoking cessation.

In terms of seeking information about smoking cessation, the results showed that Black participants preferred reviewing written materials, in contrast to White participants who mostly preferred speaking with a medical provider or participating in group discussion. While there is limited research on racial differences in preference for method of receiving health-related information, previous studies have found that Blacks are more likely to report feelings of distrust and negative experiences with doctors [44], as well as lower access and use of the healthcare system [45, 46]. One potential explanation for this finding is that continued experience with racism and discrimination may prevent Black individuals who smoke from actively seeking help from medical professionals and instead opting for self-guided research via written materials to avoid further experiences of discrimination [42]. Further investigation is needed into racial differences in preferences regarding receiving smoking cessation information, given the importance of knowledge and trust in cessation support tools. Understanding preferences for receipt of such information and incorporating them into the development of interventions can help improve smoking cessation outcomes and possibly overall engagement with the healthcare system.

Finally, the results showed that Whites reported that relationships mostly hurt their quit attempts, while Blacks mostly reported that relationships either helped or neither hurt nor helped. While there are no previous studies examining racial differences in the role of social support, partner support has been generally shown to improve smoking cessation outcomes. However, the actual impact can vary depending on factors such as the quality of the relationship and partner smoking status [47, 48] as studies have found that partners can provide negative support and add pressure or stress [49]. It is possible that the White participants in this study may have experienced negative support from their partners with regard to smoking cessation. The lack of positive or negative association between relationships and quit attempts among Black participants suggest other factors may be more salient in quit attempts among this subgroup, which may warrant further investigation.

Several key organizing themes emerged from participant narratives regardless of race, highlighting the need for their further investigation and their potential significance in the development of future interventions:

*Smoking Cigarettes Has Benefits as well as Risks.* Previous research has found that individuals associate smoking with perceived benefits such as enjoyment, stress relief, and stimulation [21] as well as risks including dependence and financial, health, time, and social risks [50]. Likewise, learning theories highlight both positive (e.g., enjoyment) and negative reinforcements (e.g., stress relief) from cigarette smoking that contribute to increased and continued smoking [51]. Despite understanding the risks well, participants continue smoking, which suggests that their perceptions of the benefits of smoking may outweigh the risks associated with smoking. Further investigation into the extent to which individuals' perceptions of the risk/reward tradeoffs associated with smoking may impact abstinence, as well as ways to influence these perceptions, may identify promising new targets for cessation interventions.

*Craving and Withdrawal Are Uncomfortable and Require Management*. Previous research has confirmed that craving and withdrawal are prominent and aversive experiences that are experienced universally, and their management is essential to cessation success [52]. While discomfort associated with cravings and withdrawal is well studied, the ways in which individuals experience and deal with them vary with a wide variety of individual and environmental factors, and the emergence of this theme highlights the importance of further investigation to understand craving/withdrawal management strategies and their role in smoking cessation outcomes.

*Individuals Have Mixed Thoughts/Feelings about Smoking Cessation*. Previous research has shown that although there are often external factors impacting individuals' perceptions and the likelihood of cessation success [53], individuals typically perceive their cessation challenges as a combination of physiological and psychological barriers [23, 53]. Participants' varying emotional reactions in considering smoking cessation may indicate their emotional connection with smoking, such that smokers may depend on nicotine to regulate their emotions (i.e., enhance positive and/or reduce negative affect). Besides emotion regulation, smokers may also associate cessation with beliefs that interventions are ineffective or unsafe, or have high financial costs [54], which may lead to a lower likelihood of seeking treatment. Further investigating and understanding the reasons why individuals may feel negatively about cessation, as well as finding potential ways to address common concerns as part of an intervention, may help improve participant readiness to quit, as well as overall cessation outcomes.

With regard to seeking help with smoking cessation, three organizing themes emerged from participant narratives: *knowledge about help for smoking cessation is common*, *individuals have strong preferences about where they find help*, and *ambivalence about cessation prevents seeking help.* These themes suggest that individuals understand the available types of assistance and have specific preferences about what type of assistance is right for them, highlighting both the availability and widespread awareness of multiple options. Furthermore, it is evident that individual characteristics and situations play a major role in participants' preferences in seeking smoking cessation assistance and, perhaps relatedly, their overall likelihood of seeking help. The emergence of these themes highlights the importance of incorporating a wide variety of resources and methods of seeking assistance into interventions, as well as further supporting the importance of investigating the factors driving attitudes toward cessation.

*Social Influences Can Be Helpful or Can Be a Barrier to Smoking Cessation*. Previous research has shown that both partner support and the general availability of support have been shown to improve short-term smoking cessation outcomes, potentially by helping ease stress and improve coping. At the same time, the presence of smokers in the individual's social networks is detrimental to abstinence [55]. Given the variability in the types of social support available to individuals and its importance in cessation success, there is a potential opportunity to incorporate training on the management of social networks and relationships in the context of smoking cessation in future interventions.

### 4.1. Limitations

Although the qualitative methods used in this study allowed for an examination of the participants' thoughts, feelings, and motivations, this approach does have certain limitations [56]. Firstly, the relatively small sample size makes it difficult to determine whether small differences between groups (e.g., 4 versus 5 participants) represent meaningful observations, especially given known limits on the generalizability of the findings and inferences obtained from qualitative research. Secondly, our sample tended to be predominantly male and older and therefore may not be fully representative of the population of cigarette smokers. Moreover, although anonymity and confidentiality of the participants' data were stressed during the informed consent process, the presence of the interviewers may have influenced the participants' responses.

Despite the limitations, the current study had several strengths, including the following: (1) matching of participants on key sociodemographic variables, including socioeconomic status, which is one of the major predictors of group differences in previous research, and (2) iterative development of the semistructured interview, including grounding in a review of the literature, as well as pilot interviews and subsequent revision of interview questions for clarity and addition of follow-up questions.

### 4.2. Conclusion

While there were no significant racial differences identified in participants' perceived consequences for smoking or thoughts and feelings about craving and withdrawal, the results suggest that there may be differences between Blacks and Whites in terms of how they manage cigarette cravings, how they prefer to receive information related to cessation, and how relationships impact their cessation efforts. The findings suggest the need for further investigation into racial differences in factors contributing to the experience of cravings and ability to control them, as well as preferences regarding receiving smoking cessation information. Further investigation into these differences will help develop our understanding and ability to address factors underlying racial disparities in smoking behavior and cessation, as well as help inform the development of future smoking cessation interventions.

## Figures and Tables

**Table 1:** Participant characteristics (*N* = 22).

Characteristic/variable	Blacks (*n* = 11) M (SD) or %	Whites (*n* = 11) M (SD) or %
Age	48.36 (8.97)	47.91 (9.57)
Partner status		
In long-term relationship	27.3%	18.2%
Dating or causal relationship	18.2%	27.3%
No relationship	54.5%	54.5%
Education in years	13.69 (1.59)	14.01 (1.90)
Income per year	40,333 (25,735)	29,000 (13,718)
Cigarettes per day	9 (5.92)	11.1 (5.281)
Age of started smoking	19.36 (4.08)	15 (2.32)
Years of smoking	26.64 (5.50)	27.73 (10.90)
FTND	3.72 (2.60)	4.20 (2.71)
Motivation to quit		
Not at all motivated	9%	36%
Not motivated	27%	9%
Motivated	45%	45%
Very motivated	18%	9%
Quit attempts in the past 12 months	2 (2.49)	0.73 (1.27)

Note: the Fagerstrom Test for Nicotine Dependence (FTND) is a standard instrument for assessing addiction to nicotine, with a total score ranging from 0 to 10.

**Table 2:** Summary of qualitative results.

Topic	Questions	Basic themes	Group differences	Organizing themes
Consequences of smoking	(i) What are the reasons you smoke? (ii) What are the benefits of smoking for you? (iii) What are the effects of smoking for you? (iv) What are the risks of smoking for you?	(i) Coping (ii) Enjoyment (iii) Dependence or habit (iv) Social pressure/connectedness (v) Taste (vi) Relaxation (vii) Financial burden (viii) Smell and appearance (ix) Health concerns	None	(i) The consequences of smoking cigarettes include benefits as well as risks

Craving and withdrawal	(i) What thoughts and/or feelings do you associate with cigarette cravings? (ii) What do you do when it is impossible to smoke? (iii) When you do not smoke for a while, how do you start thinking or feeling?	(i) Negative feelings and discomfort (ii) Positive feelings (iii) Impulsive action (iv) Use of cognitive distraction (v) Use of behavioral distraction (vi) Belief they can ignore cravings/handle cravings without distractions	(i) More White than Black participants reported that they are able to ignore or control cravings without the use of distractions	(i) Craving and withdrawal generate approach and avoidance behaviors The management of craving and withdrawal is a common experience

Smoking cessation	(i) When you think about quitting, how do you feel? (ii) What challenges do you associate with quitting smoking?	(i) Positive feelings (ii) Negative feelings (iii) Ambivalence (iv) Physical dependence (v) No substitutes (vi) Lack of interest in quitting	None	(i) Smoking cessation is characterized by significant cognitive dissonance

Help with smoking cessation	(i) How easy is it for you to find assistance with quitting smoking? (ii) What type of assistance do you think would be effective? (iii) If you were looking for more information on how to quit smoking, what would be the best way to get you that information? (iv) What is preventing you from seeking assistance in quitting? (v) There are a lot of different ways we can provide information on smoking cessation, for example, written materials, group discussion, and talking with your doctor. What do you think would suit you best?	(i) Finding assistance is easy (ii) Finding assistance is difficult (iii) Awareness of various types of assistance (iv) No need for support (v) Ambivalence about seeking help (vi) Preference for written materials (vii) Preference for speaking with a doctor (viii) Preference for participating in group discussion	(i) More Black than White participants preferred written materials (ii) More White than Black participants preferred speaking with a medical provider (iii) More White participants preferred participating in group discussion	(i) Knowledge about where to seek help for smoking cessation is common (ii) Individuals have strong preferences about what kind of help they prefer (iii) Ambivalence about cessation prevents seeking help

Social support	(i) What kind of support would you need from your social environment to help you quit smoking? (ii) What kind of help would you need from people in your life in order to quit? (iii) How do relationships help or hurt your quit attempts?	(i) Need a smoke-free environment (ii) Relationships hurt quit attempts (iii) Relationships help quit attempts (iv) Relationships neither hurt nor help quit attempts	(i) Black participants mostly reported that relationships neither hurt nor helped their quit attempts or helped their quit attempts, while White participants mostly reported that relationships hurt their quit attempts	(i) Social influences can be helpful or a barrier to smoking cessation

**Table 3:** Summary of group-specific basic themes.

Topic	Question(s)	Basic themes (Blacks)	Basic themes (Whites)
Craving and withdrawal	What do you do when it is impossible to smoke?	(i) Use of cognitive distractions (*f* = 5)**—**“take my mind off it,” “accept it,” “mentally prepare myself,” “divert my thoughts and think about something else,” “fix my mind onto something else” (ii) Use of behavioral distractions (*f* = 6)**—**“write, read, go on facebook, entertain myself,” “relax and say my prayers,” “find something else to do,” “occupy my time, read a book, watch a movie, take a nap,” “clean something up, watch some tv, go and start something that I need to do…sort of some paperwork,” “I go to sleep,” “snack on something” (iii) Belief they can ignore/handle cravings without distractions (*f* = 1)—“nothing. just bear with it”, “I accept it”	(i) Use of cognitive distractions (*f* = 3)—“do not really think about it. I know it's not in the picture, so you just do not really think about it, I cant explain how I do that, I guess you are making an adjustment in your mind where you just know its not possible to do,” “I put it out of my mind,” “I do not even think about it” (ii) Use of behavioral distractions (*f* = 5)—“I usually chew gum..I read,” “I bite my lip a lot,” “sleep…count the minutes,” “play games on my phone, whatever,” “chew gum or eat,” “read, text,” simply preoccupy myself, play games on the phone, get to eat…a latte, anything, just keep myself preoccupied doing something else” (iii) Belief they can ignore/handle cravings without distractions (*f* = 4)—“I can control it,” “It does not bother me like that these days,” “as far as substitutes for it, no I do not really do,” “Um, nothing in particular”

Help with smoking cessation	There are a lot of different ways we can provide information on smoking cessation, for example, written materials, group discussion, and talking with your doctor. What do you think would suit you best?	(i) Preference for written materials (*f* = 6)—“written instructions,” “written material if anything. Information is helpful with me. It helps combat my brain thinking that I need a cig. That's the power of information,” “written material,” “written materials.” The decision is yours. So after lots of times when you make a decision, after obtaining more information about it and lots of times you can learn more by reading about a situation and better understand it, “you can learn more by reading,” “the written material, I never tried, I'd probably just read first” (ii) Preference for speaking with a doctor (*f* = 2)—“hypnosis, I've tried them all. Yes, spoke with a doctor,” “talking with a doctor… And talking to a doctor is always good for something. I find myself talking to a doctor more often than not, lately. Just about general health, because it's a good thing to try and stay healthy,” “talking with a doctor, cause it can prescribe stuff for you to stop” (iii) Preference for participating in group discussion (*f* = 2)—“group discussions… Because it helps when you have more than one person going through the same thing youre going through” “maybe a group discussion. In a group, people are talking who are in a similar situation as yourself. And you find that it's a pool of knowledge, theres different situations, different things people have tried, you know, somethings fail and somethings work. Theres a lot of suggestions in there. I think I would get more from talking with a group than talking with a doctor”	(i) Preference for written materials (*f* = 1)—“written materials...online, because ill probably end up searching for something online. So a good place to start would be the written materials, that's just normally how I approach things. I usually like to look up things like that first and then start talking to people, like professional people or friends and family” (ii) Prefer to speak to a doctor (*f* = 5)—“probably talking to a doctor. Doctors are pretty professional and know a lot about certain things. I trust doctors, they really know which literature to go to and stuff like that,” “My doctor. She gave me these things that I suck on...I had to fight her for it. I was like...I would like to try this thing to try and stop smoking and she was like,” “no, you cannot try to stop, you have to stop smoking...,” “She just wasn't very encouraging. But I got the things...so sometimes when I do not have cigs, I'll use it,” “I would go to the doctor first,” “probably speaking with a doctor. I like getting hands on information, right there. And its pretty factual,” “Talking with a doctor. I do not need support and to talk to a doctor would be better” (iii) Prefer group discussion (*f* = 4)—“group discussion. Cause when you sit there and you are kinda confronting it, confronting your own disease. Cause you are around people that have the same problem as you, and you are hearing things that you go through from other people. And from what you say, can...I do not know...bring something out of somebody else, and you are gonna hear what you need to hear,” “group…I think its the most sympathetic and understanding place,” “group discussions. I like to talk and hear other people's view points. I need some peers like myself and wont feel so isolated and so alone in trying this,” “group discussion”

Social support	How do relationships help or hurt your quit attempts?	(i) Relationships hurt quit attempt (*f* = 1)—“Probably smoke more I guess” (ii) Relationships helped quit attempt (*f* = 4)—“help you stop smoking…yeah,” “Hurt? No. There are people that do not like smoking because they are non-smokers but they accept the fact that im a smoker because when they met me I was a smoker.” “I guess they do help whenever I do attempt to quit. I get nothing but positive feedback and support,” “By supporting me, telling me do not smoke, be there for me, if they see me smoking, take the cig away from me,” “I think it helps in a way because, you know, people who care about you, they acknowledge the harm that you are doing to yourself. And in a way they kind of appreciate the way you are starting to care about yourself a little more” (iii) Relationships neither hurt nor help quit attempts (*f* = 6)—“it depends,” “have not hurt or helped. it just…it was…the relationships were built around other things, it did not have anything to do with smoking or not smoking. Either it worked or it did not,” “Either or. it do not help or hurt,” “hurt? no,” “it has not helped or it has not hurt either”, “Varies, my wife is okay, she's known me for a very long time, as long as I do not smoke in the house there's no problem. My aunt, I cannot even smoke on her property, I cannot go outside and smoke, I cannot even smoke in front of the house on the sidewalk. It just varies with different people…my wife she called it quits a long time ago, she just gave up on me.” “You know it's just part of his character, he smokes, you know” (iv) No answer (*f* = 1)	(i) Relationships hurt quit attempt (*f* = 5)—“Relationships do not help anything… They probably do not help. Because relationships are hard, they probably want you to smoke even more. They add stress,” “Relationships? I would think they are more of a distraction. They do not help though. I do not think they do anything,” “Um, mostly hurt, like I said pretty much everyone I know smokes, so it makes it harder to actually quit. Because I know im going to be around it” (ii) Relationships helped quit attempt (*f* = 3)—“Helped…I was married at the time, and most of our problems actually had to do with smoking. Because she was totally against it and she wanted to get me to quit and we fought over it a lot of times and I said,” “okay fine I'll try, I'll do my best.” So she helped, she wanted the best for me,” “if I were in a relationship where I was living with somebody who was a non smoker and if it interfered, it was cause me to quit” (iii) Relationships neither hurt nor help quit attempts (*f* = 2)—“Neither. Like I told you, its all on me,” “Hurt or help? Well I got a new friend who buys me cigs everyday. Its been a great help… I would need a whole army...I do not know what I would need. Maybe a hypnotist, I do not know” (iv) Did not know how to respond (*f* = 1)—“Well lately, everyone has alienated me, so its just me. I really do not even have an answer to that right now, you know?”

Note: this table only includes basic themes where participants' responses differed per group. All basic themes, including those where there was no notable difference in response based on groups, are reported in Table 2. *f* is frequency.

## Data Availability

Requests to access these datasets should be directed to the corresponding author. Data is not publicly available.
